# Correlation between serum sex hormone-binding globulin levels and nutrition indicators and malnutrition exposure risk in men and postmenopausal women with type 2 diabetes

**DOI:** 10.1186/s12902-024-01653-x

**Published:** 2024-07-17

**Authors:** Jinxin Lin, Weiming Wu, Yifu Weng, Yingru Lan, Yuqiong Wen, Shuiqing Lai, Xiaoying Fu, Jian Kuang, Haixia Guan, Hongmei Chen

**Affiliations:** 1 Department of Endocrinology, Guangdong Provincial People’s Hospital (Guangdong Academy of Medical Sciences), Southern Medical University, No.106 Zhongshan Second Road, Guangzhou, 510080 P.R. China; 2 Shantou University Medical College, Shantou, 515041 P.R. China; 3 Xiehe Shenzhen Hospital, Huazhong University of Science and Technology, Shenzhen, 518051 P.R. China

**Keywords:** Sex hormone-binding globulin, Malnutrition, Type 2 diabetes mellitus, Cross-sectional study

## Abstract

**Background:**

This study sought to investigate the correlation between serum sex hormone-binding globulin (SHBG) levels and nutrition indicators and the malnutrition exposure risk in men and postmenopausal women with type 2 diabetes mellitus (T2DM).

**Methods:**

A cross-sectional analysis was conducted, involving patients diagnosed with T2DM at the Guangdong Provincial People’s Hospital between May 2018 and December 2019.

**Results:**

The study comprised 551 participants (363 men, mean age of 55.55 ± 11.57 years), among whom 167 (30.31%) were classified as with malnutrition exposure risk (GNRI ≤ 98). Multivariable logistic regression analysis revealed that SHBG (OR = 1.04, 95% CI: 1.02–1.05, *P* < 0.001), glycated hemoglobin (OR = 1.36, 95% CI: 1.22–1.51, *P* < 0.001), hemoglobin (OR = 0.96, 95% CI: 0.94–0.97, *P* < 0.001), and non-alcoholic fatty liver disease (OR = 0.41, 95% CI: 0.23–0.73, *P* < 0.003) were independently associated with the malnutrition exposure risk. SHBG was inversely correlated with body mass index (males: *r* = -0.34; postmenopausal females: *r* = -0.22), albumin (males: *r* = -0.30; postmenopausal females: *r* = -0.20), transferrin (males: *r* = -0.28; postmenopausal females: *r* = -0.19), and prealbumin (males: *r* = -0.35; postmenopausal females: *r* = -0.30) (all *P* < 0.05).

**Conclusions:**

Serum SHBG levels are correlated with nutritional indicators and the risk of malnutrition in men and postmenopausal women with T2DM. A multicenter prospective study is imperative to verify this result in the future.

**Supplementary Information:**

The online version contains supplementary material available at 10.1186/s12902-024-01653-x.

## Background

Type 2 diabetes mellitus (T2DM) is a prevalent endocrine disorder characterized by varying degrees of insulin resistance and deficiency, leading to hyperglycemia [1]. The potential complications associated with T2DM encompass cardiovascular disease, neuropathy, nephropathy, retinopathy, and elevated mortality rates [2]. Moreover, individuals with T2DM are particularly susceptible to malnutrition, notably among older adults and those suffering from chronic conditions [3–5]. This malnourished state frequently presents as deficits in essential micronutrients and protein, markedly affecting these individuals’ quality of life and prognosis [6–9]. The prevalence of malnutrition is notable amongst hospitalized patients with T2DM [10], highlighting the critical importance of addressing malnutrition in the management of T2DM.

Traditionally, body weight, BMI, serum albumin, prealbumin, and transferrin have been considered as nutritional indicators [11]. Sex hormone-binding globulin (SHBG) is a glycated, homodimer transport protein primarily produced by the liver, displaying a higher affinity for androgens such as testosterone and dihydrotestosterone over estradiol [12]. Previous research has identified a link between reduced serum SHBG levels and the development of various cardiometabolic conditions in T2DM patients. Studies have demonstrated that postmenopausal females are at increased risk for metabolic diseases as they go through menopause. It is known that there are gender-based differences in SHBG expression and premenopausal women experience fluctuations in SHBG levels during the menstrual cycle [13]. However, recent studies have shown that serum SHBG levels were linked to the nutritional status of individuals with primary infertility [14], anorexia nervosa [15], kwashiorkor [16], and polycystic ovary syndrome [17]. Notably, SHBG levels are increased in conditions such as anorexia nervosa and kwashiorkor [16]. Studies have demonstrated an inverse relationship between SHBG levels and weight, where elevated SHBG levels lead to reduced testosterone bioavailability [18]. Some studies have suggested that elevated serum SHBG levels, alongside reduced total testosterone levels, are associated with increased mortality rates [19], malnutrition, sarcopenia, and frailty in males with T2DM. These observations prompt further investigation into the relationship between serum SHBG levels and the nutritional status of patients.

Therefore, the relationship of SHBG levels and nutritional risks in men and postmenopausal women with T2DM remains unclear. Accordingly, this cross-sectional study was designed to elucidate the correlation between serum SHBG levels and nutritional indicators and the risk of malnutrition in men and postmenopausal women with T2DM.

## Methods

### Study design and participants

This cross-sectional study was conducted at the National Metabolic Management Center (MMC) within the Guangdong Provincial People’s Hospital (GDPH), a subsidiary of the China MMC network, between May 2018 and December 2019. The study was registered (ClinicalTrials.gov identifier: NCT03811470. First posted on January 22, 2019. Last update posted on June 6, 2022. Actual study started on May 31, 2017). The MMC represents a standardized management system inaugurated in 2016 to facilitate efficient diagnosis, treatment, and sustained follow-up of metabolic disorders [20]. The study received approval from the research ethics committees of Guangdong Provincial People’s Hospital (reference #GDREC2012067H [R1]) and Ruijin Hospital, adhering to the principles outlined in the Declaration of Helsinki. Before participation, all participants rendered written informed consent.

The inclusion criteria encompassed (1) being male or a postmenopausal female, (2) aged between 18 and 75 years, and (3) having a diagnosis of T2DM in accordance with the 1999 World Health Organization criteria [21], either newly diagnosed or under stable treatment for the preceding three months. The exclusion criteria specified (1) severe cardiac, hepatic, renal diseases, thyroid dysfunction or cancer, (2) a protracted history of alcoholism, (3) drug misuse, (4) an expected lifespan of less than five years, (5) found to carry the rs6257 or rs6259 variant allele in the SHBG gene through genotyping or (6) the presence of acquired immune deficiency syndrome, syphilis, or infectious conditions such as viral hepatitis or tuberculosis in their active stages at the time of enrolment.

### Procedures

The procedures adhered to the MMC’s protocol. All participants completed a standardized questionnaire and undergoing comprehensive clinical and laboratory assessments upon registration. Data were meticulously gathered and documented by trained personnel using the MMC’s bespoke electronic medical record system [20]. The collected clinical data and laboratory findings, extracted from medical records, included demographic and health-related metrics such as age, gender, weight, height, body mass index (BMI), albumin levels, duration of diabetes, SHBG, prealbumin, transferrin, glycated hemoglobin (HbA1c), C-peptide, triglycerides, cholesterol, low-density lipoprotein cholesterol (LDL-C), uric acid, hemoglobin, insulin-like growth factor (IGF)-1, aspartate aminotransferase (AST), alanine aminotransferase (ALT), creatinine, estimated glomerular filtration rate (eGFR), the presence of non-alcoholic fatty liver disease (NAFLD), and cardiovascular diseases (CVD). The BMI was calculated by dividing the body weight (in kilograms) by the square of the height (in meters). Laboratory analyses, including serum cholesterol, triglycerides, and LDL-C, were quantified via enzymatic oxidation methods at the hospital’s central laboratory. Glycosylated hemoglobin levels were ascertained through high-pressure liquid chromatography, whereas serum SHBG concentrations were measured employing an enzyme-linked immunosorbent assay (Guangzhou Kingmed Diagnostics, Guangzhou, China). The diagnosis of hypertension or CVD was confirmed from medical records, and NAFLD was diagnosed through abdominal ultrasonography.

The risk of malnutrition exposure was evaluated using the Geriatric Nutritional Risk Index (GNRI). This validated prognostic model expands upon the traditional Nutritional Risk Index (NRI) by incorporating critical biological variables such as weight, height, and serum albumin levels. The GNRI calculation employed the formula: 1.489 × albumin concentration (g/L) + 41.7 × (actual weight / ideal weight), wherein the ideal weight was ascertained using the Lorentz formula: for men, (height – 100) – (height – 150) / 4, and for women, (height – 100) – (height – 150) / 2.5. A GNRI value of ≤ 98 indicated a risk of malnutrition [22]. Participants were stratified into categories based on their GNRI scores: no risk (GNRI > 98), low risk (GNRI 92–98), and moderate to major risk (GNRI < 92). Subsequently, patients were classified into without malnutrition exposure risk (GNRI > 98) and with malnutrition exposure risk (GNRI ≤ 98) groups.

DNA extraction from patients’ blood cells was carried out utilizing the Genome Extraction Kit (Tiangen Biochemical Technology, Beijing, China). The DNA concentration and purity were quantified via the Nanodrop 2000 Spectrophotometer (Thermo Fisher Scientific, Waltham, MA, USA), with DNA samples exhibiting concentrations between 40 and 120 ng/µl and purity (OD260/OD280) ratios ranging from 1.6 to 1.8. Genotyping was conducted using the TaqMan-MGB probe assay (Life Technologies Co., Grand Island, NY, USA) to identify specific single-nucleotide polymorphisms (SNPs) in the SHBG gene, notably rs6257 and rs6259, which have been previously reported to influence serum SHBG levels [23–26]. Participants with the variant alleles for rs6257 (CC or CT) or rs6259 (AA or AG) were omitted from the final analysis to mitigate potential confounding effects on serum SHBG levels in individuals with T2DM [25].

### Sample size

The required sample size for this study was computed using the PASS 15.0.5 software (NCSS LLC, Kaysville, UT, USA), considering the existing data on malnutrition prevalence among T2DM patients [5, 27, 28]. With an alpha of 0.05, a precision of 0.08, and a prevalence rate of 0.3, the minimum sample size necessary to estimate the prevalence of malnutrition in the T2DM cohort was 527 participants.

### Statistical analysis

Statistical analyses were performed utilizing SPSS 25 (IBM, Armonk, NY, USA) and GraphPad Prism 9 (GraphPad Software Inc., San Diego, CA, USA). Continuous variables are expressed as mean ± standard deviation (SD) for normally distributed data or median and interquartile range (IQR) for data with skewed distribution. Categorical variables are presented as frequencies and percentages (n [%]). The continuous variables were analyzed using the student’s t-test for normally distributed data and the Mann-Whitney U-test for non-normally distributed data. Categorical variables were evaluated using the chi-squared test. The independent relationship between SHBG levels and the risk of malnutrition exposure was determined through binary logistic regression. Variables with *P* < 0.05 in univariable analysis were subsequently entered into multivariable logistic regression analysis. Odds ratios (ORs) and their 95% confidence intervals (CIs) were reported. The correlation between serum SHBG levels and nutritional indices was assessed via Spearman correlation analysis. Subgroup analyses were conducted for men and postmenopausal women. *P*-values of less than 0.05 were deemed to indicate statistical significance.

## Results

### Characteristics of the participants

**Fig. 1:**
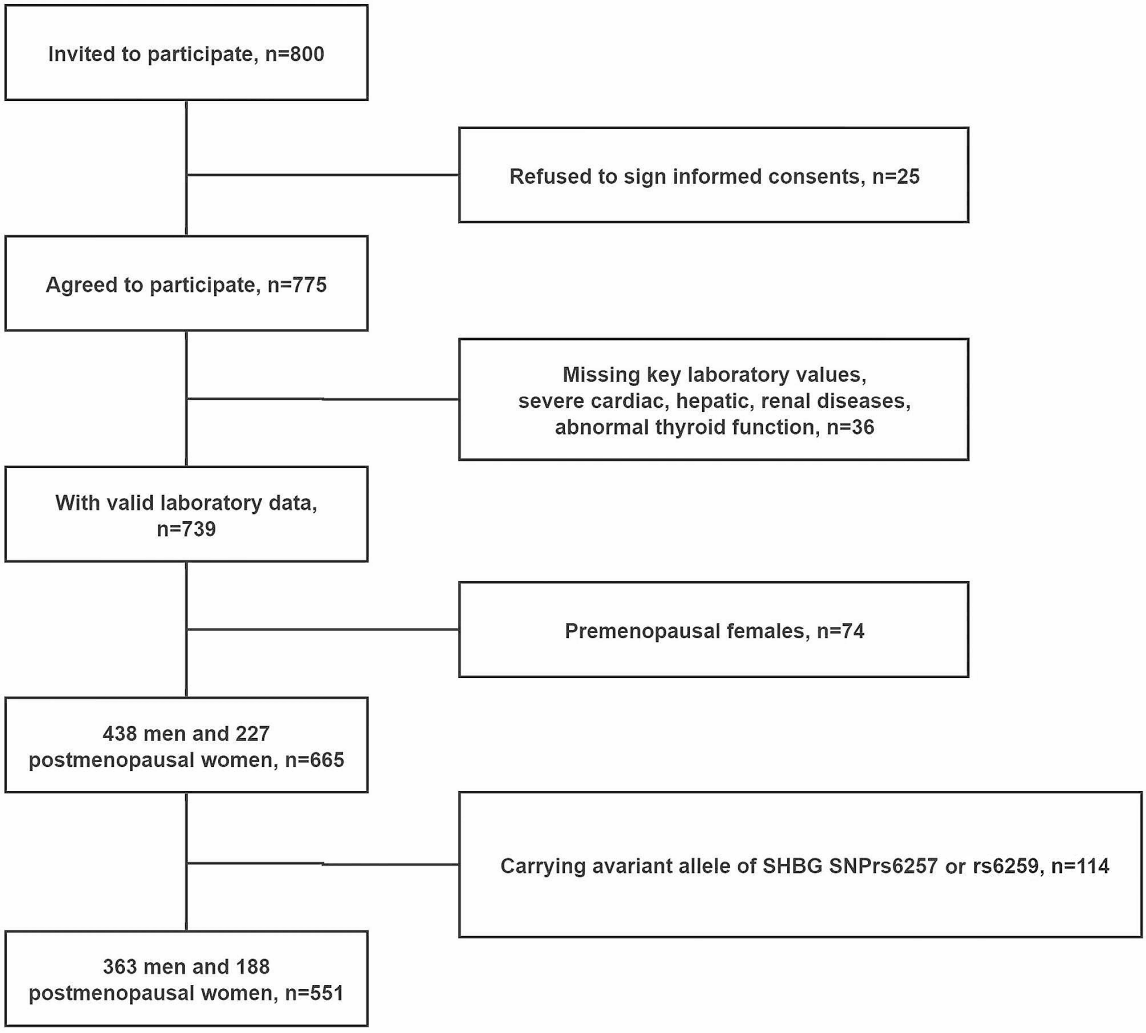
Participant flowchart

Out of 800 men and postmenopausal women with T2DM solicited for participation, 25 declined, 36 lacked essential laboratory data or had severe cardiac, hepatic, renal disease or thyroid dysfunction, 74 were premenopausal women, and 114 possessed a variant SHBG SNP at rs6257 or rs6259. Consequently, 551 participants (363 men [65.88%]; mean age 55.55 ± 11.57 years) were enrolled (Fig. 1). Within this cohort, 384 individuals (69.69%) had a GNRI above 98, categorizing them as without malnutrition exposure risk, while 167 (30.31%) had a GNRI of 98 or below, placing them in the with malnutrition exposure risk category. Compared to their without malnutrition exposure risk counterparts, the with malnutrition exposure risk group exhibited significantly elevated levels of SHBG and HbA1c and reduced C-peptide, triglycerides, uric acid, hemoglobin, IGF-1, AST, ALT, weight, BMI, albumin, prealbumin, and transferrin. The patients in with malnutrition exposure risk group were less likely to be diagnosed with NAFLD, CVD, retinopathy, and hypertension (all *P* < 0.05) (Table 1).

**Table 1:** Baseline characteristics of the participants

Variables	Total (*n* = 551)	Without malnutrition exposure risk (*n* = 384)	With malnutrition exposure risk (*n* = 167)	*P*
Age, years	55.55 ± 11.57	55.49 ± 11.29	55.68 ± 12.22	0.859
Sex, n (%)				0.555
Male	363 (65.88)	256 (66.67)	107 (64.07)	
Postmenopausal female	188 (34.12)	128 (33.33)	60 (35.93)	
Diabetes duration, months	84.00 (14.25–156.00)	84.00 (13.00-151.50)	120.00 (24.00-180.00)	0.107
SHBG, nmol/L	35.65 ± 19.48	30.73 ± 15.14	46.98 ± 23.30	< 0.001
HbA1c, %	9.82 ± 2.52	9.40 ± 2.20	10.82 ± 2.91	< 0.001
C-peptide, nmol/L	0.59 (0.37–0.88)	0.66 (0.43–0.95)	0.40 (0.23–0.67)	< 0.001
Triglyceride, mmol/L	1.51 (1.07–2.22)	1.60 (1.13–2.51)	1.26 (0.87–1.82)	< 0.001
Cholesterol, mmol/L	4.97 (4.11–5.90)	4.92 (4.20–5.79)	5.00 (4.00-6.02)	0.790
LDL-C, mmol/L	3.35 ± 0.97	3.32 ± 0.86	3.40 ± 1.19	0.470
Uric acid, µmol/L	384.59 ± 109.68	398.64 ± 105.99	352.29 ± 111.51	< 0.001
Hemoglobin, g/L	134.31 ± 19.09	138.04 ± 16.99	125.70 ± 20.86	< 0.001
IGF-1, ng/mL	155.40 ± 72.89	162.01 ± 72.61	140.15 ± 71.46	0.001
AST, U/L	19.00 (15.00–24.00)	20.00 (16.00–24.00)	17.00 (14.00-23.50)	0.004
ALT, U/L	20.00 (14.00–28.00)	21.00 (15.00–30.00)	17.00 (13.00-24.67)	< 0.001
Creatinine, µmol/L	67.15 (56.52–84.68)	67.35 (57.60-83.35)	65.82 (52.80-90.34)	0.718
eGFR, (mL/min/1.73 m^2^)	103.84 (80.49-126.36)	103.56 (82.96-125.47)	104.69 (72.22-131.99)	0.998
Weight, kg	66.48 ± 12.46	69.98 ± 11.74	58.42 ± 10.14	< 0.001
Height, cm	164.59 ± 8.04	164.86 ± 8.07	163.97 ± 7.95	0.232
BMI, kg/m^2^	24.46 ± 3.73	25.66 ± 3.31	21.64 ± 3.09	< 0.001
Albumin, g/L	37.54 ± 4.63	39.43 ± 3.14	33.19 ± 4.59	< 0.001
Prealbumin, mg/L	233.44 ± 62.59	251.19 ± 53.01	194.70 ± 64.53	< 0.001
Transferrin, mg/dl	1.99 ± 0.36	2.10 ± 0.30	1.75 ± 0.36	< 0.001
NAFLD, n (%)	221 (40.11)	192 (50.00)	29 (17.37)	< 0.001
CVD, n (%)	178 (32.3)	142 (36.98)	36 (21.56)	< 0.001
Hypertension, n (%)	263 (47.73)	194 (50.52)	69 (41.32)	0.047
Retinopathy, n (%)	154 (27.95)	90 (23.44)	64 (38.32)	< 0.001
ABI, n (%)	118 (21.42)	85 (22.14)	33 (19.76)	0.532

Continuous variables are reported as mean ± SD for normally distributed variables or as the median (IQR) for skewed variables, while categorical variables are represented as numbers (proportions)

BMI: body mass index; HbA1c: hemoglobin A1c; SHBG: sex hormone-binding globulin; ALT: alanine aminotransferase; AST: aspartate aminotransferase; HDL-C: high-density lipoprotein cholesterol; eGFR: estimated glomerular filtration rate; IGF-1: insulin-like growth factor-1; CVD: cardiovascular disease; ABI: ankle-brachial index; NA: not available

### Association between serum SHBG levels and malnutrition exposure risk

Multivariable logistic regression analysis indicated that SHBG (OR = 1.04, 95% CI: 1.02–1.05, *P* < 0.001), HbA1c (OR = 1.36, 95% CI: 1.22–1.51, *P* < 0.001), hemoglobin (OR = 0.96, 95% CI: 0.94–0.97, *P* < 0.001), and NAFLD (OR = 0.41, 95% CI: 0.23–0.73, *P* = 0.003) were independently associated with the risk of malnutrition exposure (Table 2).

**Table 2:** Univariable and multivariable analysis of the malnutrition exposure risk among all participants

Variables	Univariable analysis		Multivariable analysis	
	OR (95%CI)	*P*	OR (95%CI)	*P*
Age	1.00 (0.99–1.01)	0.859		
Sex	1.12 (0.77–1.64)	0.555		
SHBG	1.05 (1.04–1.06)	< 0.001	1.04 (1.02–1.05)	< 0.001
HbA1c	1.26 (1.16–1.36)	< 0.001	1.36 (1.22–1.51)	< 0.001
C-peptide	0.27 (0.16–0.46)	< 0.001	0.84 (0.47–1.50)	0.547
Triglyceride	0.76 (0.65–0.90)	0.001	0.92 (0.76–1.10)	0.362
Hemoglobin	0.97 (0.95–0.98)	< 0.001	0.96 (0.94–0.97)	< 0.001
Uric acid	0.99 (0.99–0.99)	< 0.001	1.00 (1.00–1.00)	0.420
IGF-1	0.99 (0.99–0.99)	0.002	1.00 (1.00–1.00)	0.500
AST	1.00 (0.98–1.01)	0.847		
ALT	0.98 (0.97–0.99)	0.008	0.98 (0.97-1.00)	0.115
NAFLD	0.21 (0.13–0.33)	< 0.001	0.41 (0.23–0.73)	0.003
CVD	0.47 (0.31–0.71)	< 0.001	0.59 (0.33–1.04)	0.066
Hypertension	0.69 (0.48–0.99)	0.047	0.62 (0.37–1.06)	0.082
Retinopathy	1.86 (1.30–2.66)	< 0.001	1.06 (0.65–1.74)	0.810

SHBG: sex hormone-binding globulin; HbA1c: hemoglobin A1c; IGF-1: insulin-like growth factor-1; AST: aspartate aminotransferase; ALT: alanine aminotransferase; NAFLD: non-alcoholic fatty liver disease; CVD: cardiovascular disease

### Correlations between serum SHBG levels and nutritional indicators

In male subjects, there was a negative correlation between BMI and serum SHBG levels (*r* = -0.34, *P* < 0.01), as well as between serum albumin and SHBG (*r* = -0.30, *P* < 0.01), serum transferrin and SHBG (*r* = -0.28, *P* < 0.01), and serum prealbumin and SHBG (*r* = -0.35, *P* < 0.01). Comparable findings were observed in postmenopausal females, where significant negative correlations were noted between BMI and serum SHBG (*r* = -0.22, *P* < 0.01), serum albumin and SHBG (*r* = -0.20, *P* < 0.001), serum transferrin and SHBG (*r* = -0.19, *P* < 0.05), and serum prealbumin and SHBG (*r* = -0.30, *P* < 0.01) (Table 3).

**Table 3:** Spearman correlation coefficients of SHBG and nutritional indicators

		BMI	Albumin	Transferrin	Prealbumin
SHBG	Male	-0.34**	-0.30**	-0.28**	-0.35**
	Postmenopausal female	-0.22**	-0.20**	-0.19*	-0.30**

* *P* < 0.05, ** *P* < 0.01

SHBG: sex hormone-binding globulin; BMI: body mass index

### Subgroup analysis

In male participants, significant disparities were observed between the without malnutrition exposure risk and with malnutrition exposure risk cohorts concerning SHBG (28.94 ± 13.43 vs. 46.62 ± 24.16 nmol/L, *P* < 0.001), HbA1c (9.49 ± 2.34 vs. 10.97 ± 3.16%, *P* < 0.001), C-peptide (0.63 [0.40–0.94] vs. 0.32 [0.20–0.57] nmol/L, *P* < 0.001), uric acid (409.28 ± 101.00 vs. 362.48 ± 108.63 µmol/L, *P* < 0.001), hemoglobin (143.46 ± 15.95 vs. 131.69 ± 20.08 g/L, *P* < 0.001), IGF-1 (168.17 ± 70.44 vs. 141.89 ± 78.54 ng/mL, *P* = 0.002), ALT (21.00 [15.00–30.00] vs. 19.00 [13.00–25.00] µmol/L, *P* = 0.013), triglycerides (1.54 [1.10–2.13] vs. 1.10 [0.83–1.55] mmol/L, *P* < 0.001), and the prevalence of NAFLD, CVD, or hypertension (Supplementary Table S1). Multivariable logistic regression analysis included the variables demonstrating a P level < 0.05 in the univariable analysis, and indicated that serum SHBG levels (OR = 1.04, 95% CI: 1.02–1.06, *P* < 0.001), HbA1c (OR = 1.28, 95% CI: 1.11–1.46, *P* < 0.001), and hemoglobin levels (OR = 0.96, 95% CI: 0.94–0.98, *P* < 0.001) were independently associated with the risk of malnutrition exposure (Table 4).

**Table 4:** Univariable and multivariable analysis of the malnutrition exposure risk among males

Variables	Univariable analysis		Multivariable analysis	
	OR (95%CI)	*P*	OR (95%CI)	*P*
Age	0.99 (0.97–1.01)	0.277		
SHBG	1.06 (1.04–1.07)	< 0.001	1.04 (1.02–1.06)	< 0.001
HbA1c	1.30 (1.17–1.44)	< 0.001	1.28 (1.11–1.46)	< 0.001
C-peptide	0.06 (0.02–0.17)	< 0.001	0.46 (0.15–1.46)	0.190
Triglyceride	0.72 (0.55–0.94)	0.014	0.93 (0.69–1.23)	0.598
Hemoglobin	0.97 (0.95–0.98)	< 0.001	0.96 (0.94–0.98)	< 0.001
IGF-1	0.99 (0.99–0.99)	0.007	1.00 (0.99-1.00)	0.525
ALT	1.00 (0.98–1.02)	0.885		
Uric acid	0.99 (0.99–0.99)	< 0.001	1.00 (1.00–1.00)	0.756
NAFLD	0.25 (0.14–0.46)	< 0.001	0.57 (0.26–1.25)	0.163
CVD	0.34 (0.18–0.66)	0.001	0.54 (0.23–1.27)	0.156
Retinopathy	1.37 (0.78–2.42)	0.272		
Hypertension	0.54 (0.32–0.93)	0.026	0.92 (0.42–2.01)	0.835

SHBG: sex hormone-binding globulin; HbA1c: hemoglobin A1c; HDL-C: high-density lipoprotein cholesterol; IGF-1: insulin-like growth factor-1; eGFR: estimated glomerular filtration rate; NAFLD: non-alcoholic fatty liver disease; CVD: cardiovascular disease

For postmenopausal women, notable differences were observed between the without malnutrition exposure risk and with malnutrition exposure risk groups in terms of SHBG (34.30 ± 17.61 vs. 47.61 ± 21.87 nmol/L, *P* < 0.001), HbA1c (9.23 ± 1.92 vs. 10.55 ± 2.41%, *P* < 0.001), C-peptide (0.73 [0.50–0.96] vs. 0.55 [0.37–0.88] nmol/L, *P* = 0.005), uric acid (377.45 ± 112.75 vs. 332.86 ± 115.34 µmol/L, *P* = 0.016), hemoglobin (127.20 ± 13.50 vs. 115.12 ± 17.90 g/L, *P* < 0.001), AST (20.00 [17.00-26.25] vs. 16.50 [13.00–21.00] U/L, *P* < 0.001), ALT (20.00 [15.00-30.50] vs. 16.00 [10.75–23.25] U/L, *P* < 0.001), triglycerides (1.92 [1.23–2.81] vs. 1.60 [1.14–2.02] mmol/L, *P* = 0.020) levels, and the presence of NAFLD or retinopathy (Supplementary Table S2). Multivariable logistic regression analysis included the variables demonstrating a P level < 0.05 in the univariable analysis, and demonstrated that serum SHBG levels (OR = 1.04, 95% CI: 1.01–1.06, *P* = 0.004), HbA1c levels (OR = 1.58, 95% CI: 1.25-2.00, *P* < 0.001), hemoglobin levels (OR = 0.90, 95% CI: 0.86–0.95, *P* < 0.001), AST (OR = 0.93, 95% CI: 0.90–0.97, *P* < 0.001), and NAFLD (OR = 0.23, 95% CI: 0.08–0.68, *P* = 0.008) were independently associated with the risk of malnutrition exposure (Table 5).

**Table 5:** Univariable and multivariable analysis of the malnutrition exposure risk among postmenopausal females

Variables	Univariable analysis		Multivariable analysis	
	OR (95%CI)	*P*	OR (95%CI)	*P*
Age	0.99 (0.95–1.04)	0.684		
SHBG	1.03 (1.02–1.05)	< 0.001	1.04 (1.01–1.06)	0.004
HbA1c	1.34 (1.15–1.57)	< 0.001	1.58 (1.25-2.00)	< 0.001
C-peptide	0.43 (0.20–0.90)	0.025	1.11 (0.50–2.48)	0.792
Hemoglobin	0.93 (0.90–0.96)	< 0.001	0.90 (0.86–0.95)	< 0.001
AST	0.95 (0.93–0.97)	< 0.001	0.93 (0.90–0.97)	< 0.001
ALT	0.94 (0.90–0.98)	0.005	0.94 (0.85–1.04)	0.244
Uric acid	0.99 (0.99–0.99)	0.019	1.00 (0.99-1.00)	0.056
Triglyceride	0.95 (0.93–0.99)	0.004	1.00 (0.93–1.07)	0.919
NAFLD	0.14 (0.06–0.31)	< 0.001	0.23 (0.08–0.68)	0.008
Retinopathy	2.59 (1.36–4.91)	0.004	1.04 (0.38–2.80)	0.944

SHBG: sex hormone-binding globulin; HbA1c: hemoglobin A1c; AST: aspartate aminotransferase; ALT alanine aminotransferase; NAFLD: non-alcoholic fatty liver disease

Furthermore, an upward trend in SHBG levels was observed with increasing risk of malnutrition exposure in both males (29.14 ± 13.38, 41.52 ± 21.58, and 49.99 ± 25.21 nmol/L, respectively) and postmenopausal women (32.82 ± 16.51, 43.92 ± 19.32, and 50.47 ± 26.49 nmol/L, respectively) (*P* < 0.05) (Supplementary Figure S1B).

## Discussion

The findings imply that males and postmenopausal females with T2DM may associated with a significant risk of malnutrition exposure, with elevated serum SHBG levels being independently correlated with an increased malnutrition exposure risk in these demographics. Notably, serum SHBG levels were inversely related to various nutritional indices, including BMI, serum albumin, prealbumin, and transferrin. These results could facilitate the identification of patients at elevated risk of malnutrition exposure.

T2DM is recognized as a globally prevalent chronic disease [29]. Malnutrition is frequently observed in hospitalized individuals [30]. In this study, the malnutrition exposure risk was defined by a GNRI ≤ 98, with 30.98% of patients with T2DM experiencing the malnutrition exposure risk. Previous research in Switzerland, utilizing the NRS 2002 score, indicated malnutrition prevalence rates ranging from 18.2 to 27.8% among hospitalized patients [27, 28]. In China, malnutrition prevalence among patients with abnormal glycemic status and coronary artery disease varied from 8 to 57%, based on four nutritional assessment tools [5]. These patients demonstrated an increased long-term risk of all-cause mortality. T2DM patients were more susceptible to conditions such as multivessel coronary artery disease, acute myocardial infarction, atrial fibrillation, congestive heart failure, hypertension, stroke, and anemia. Wen Wei et al. had shown malnutrition may associate with the increase of coronary artery disease in T2DM patients [31]. Additionally, studies have indicated that malnutrition might contribute to the increase of cardiometabolic conditions among postmenopausal women with T2DM [9, 32].

Malnutrition is frequently associated with poverty, social isolation and substance misuse [33]. In the elderly population, barriers to adequate nutrition may arise from mobility impairment, cognitive decline, and functional limitations [34]. Among older adults with T2DM, a higher prevalence and vulnerability to malnutrition has been found compared to those without T2DM [35]. This heightened risk can be partially ascribed to autonomic neuropathy associated with T2DM, which presents as symptoms such as anorexia and gastroparesis [4, 36]. Additionally, the effects of antihyperglycemic medications and strict dietary restrictions or low caloric intake, intended to regulate blood glucose levels, may also contribute to the development of malnutrition [37, 38].

SHBG, predominantly synthesized in the liver, is pivotal in regulating testosterone and other sex hormones by controlling their transport, tissue distribution, bioavailability, and metabolism. There have been studies indicating the association between dietary intake and serum SHBG levels. Allen et al. [39] and Longcope et al. [18] identified a positive association between dietary fiber intake and SHBG levels, while a negative correlation was observed with the consumption of animal fats. Moreover, Samimisedeh et al. [40] found significantly higher SHBG levels in vegetarian men than omnivores. This study has also noted a relationship between elevated serum SHBG levels and an increased risk of malnutrition in men and postmenopausal women with T2DM. Similarly, Boeri et al. [14] discovered an inverse relationship between serum SHBG levels and BMI. The study observed inverse correlations between serum SHBG levels and various nutritional indices, including BMI, serum albumin, prealbumin, and transferrin, with lower levels of serum albumin, prealbumin, and transferrin, alongside higher serum SHBG levels, being indicative of the risk of malnutrition in individuals with T2DM. Further gender-specific studies are necessary to explore the impact of gender and endogenous sex hormone levels on the risk of malnutrition in patients with T2DM.

The impact of malnutrition on fertility may elucidate the findings. The fraction of sex hormones bound to SHBG is deemed biologically inert owing to SHBG’s pronounced binding affinity. In accordance with the Free Hormone Hypothesis, which postulates that solely the unbound or free fraction holds biological activity within target tissues, augmented levels of circulating SHBG may diminish the presence of biologically active sex hormones [41]. SHBG chiefly governs the partition of circulating sex hormones into their bound and unbound components, particularly in males [12]. Consequently, an individual’s testosterone bioavailability would be relatively diminished with elevated circulating SHBG levels. It is conceivable that during periods of malnutrition, the human organism may deploy a strategy to hinder the reproductive process, which is metabolically demanding. The upregulation of circulating SHBG levels might serve to downregulate active sex hormones, thereby impeding the reproductive process. Therefore, the detection of elevated SHBG levels in undernourished patients potentially suggests the existence of a biological self-protective mechanism during stressful periods, necessitating further inquiry. Another hypothesis posits that testosterone serves as an anabolic hormone. Consequently, reduced bioavailable testosterone due to elevated SHBG levels might contribute to malnutrition, sarcopenia, and frailty.

One notable strength of our study was the incorporation of SHBG SNPs in our analysis. Considering the potential impact of gene polymorphisms on serum SHBG levels, individuals carrying the rs6257 or rs6259 variant allele were excluded from our investigation. Our findings revealed a significant association between elevated serum SHBG levels and heightened risk of malnutrition exposure in males and postmenopausal women with T2DM. Additionally, we observed inverse relationships between serum SHBG levels and various nutritional biomarkers such as BMI, serum albumin, prealbumin, and transferrin. These findings might enhance the clinical significance of SHBG in the assessment of malnutrition.

However, this study has several limitations. Firstly, this study adopted a cross-sectional design, precluding the establishment of causality. Despite stringent quality control measures, data from electronic health records may be biased by misdiagnoses and inconsistent coding, and using cross-sectional data implies that the findings lack validation from longitudinal cohort data. Secondly, the associations in premenopausal females were not explored, considering the potential interference from the menstrual cycle on SHBG levels. Therefore, the generalizability of the study is limited. Thirdly, other malnutrition indices, such as the PNI and the NRI, could not be appraised due to the unavailability of requisite data. Additionally, there are several other potential confounding variables that were not considered in the study, such as protein intake, educational and financial situation, and medication usage. Consequently, although an association between elevated serum SHBG levels and an increased malnutrition exposure risk in patients with T2DM was discerned, further investigation that includes a broader demographic sample and considers additional confounding variables is imperative to comprehend this relationship.

## Conclusions

In conclusion, this study underscores the significant risk of malnutrition exposure in men and postmenopausal women with T2DM. SHBG levels exhibit correlations with nutritional indicators, and elevated serum SHBG levels are linked with an augmented risk of malnutrition in male and postmenopausal female T2DM patients. A multicenter prospective study is imperative to verify this result in the future.

### Electronic supplementary material

Below is the link to the electronic supplementary material.

Supplementary Material 1

Supplementary Material 2

## Data Availability

All data generated or analysed during this study are included in this published article and its Supplementary Information files.

## Abbreviations

SHBG: Sex hormone-binding globulin
T2DM: Type 2 diabetes mellitus
BMI: Body mass index
HbA1c: Glycated hemoglobin
LDL-C: Low-density lipoprotein cholesterol
IGF: Insulin-like growth factor
AST: Aspartate aminotransferase
ALT: Alanine aminotransferase
eGFR: Estimated glomerular filtration rate
NAFLD: Non-alcoholic fatty liver disease
CVD: Cardiovascular diseases
