# Supplementary material for: Correlation between serum sex hormone-binding globulin levels and nutrition indicators and malnutrition exposure risk in men and postmenopausal women with type 2 diabetes
Source: BMC Endocr Disord. 2024 Jul 17;24:117. doi: 10.1186/s12902-024-01653-x (PMC11253498; doi:10.1186/s12902-024-01653-x)
Supplement: Supplementary file 1 — Supplementary Material 1 [file 12902_2024_1653_MOESM1_ESM.docx]

**Patient Information Questionnaire**

Source : Guangdong Provincial Medical Research Project (2012018)

Title: "The mechanism of hypogonadism and insulin resistance in T2DM patients",

Informed Consent Version ID (GDPH (No. GDREC2012067H[R1]))

| Patient's name  (Initial acronym) |  | | Gender Identity | Male ○ Female ○ | |
| --- | --- | --- | --- | --- | --- |
| Condition/Diagnosis |  | | | | |
| Case ID |  | | | | |
| MMC ID |  | | | | |
| Have the Questionnaires been completed? | No | | Yes |  | |
| Biochemical Testing Date： | | | | | |
|  | | Estimated value | | | Unit |
| Prealbumin | |  | | | mg /L |
| Transferr in | |  | | | mg /dl |
| DNA Testing:  SHBG SNPs | | | | | |
