# Supplementary material for: Correlation between serum sex hormone-binding globulin levels and nutrition indicators and malnutrition exposure risk in men and postmenopausal women with type 2 diabetes
Source: BMC Endocr Disord. 2024 Jul 17;24:117. doi: 10.1186/s12902-024-01653-x (PMC11253498; doi:10.1186/s12902-024-01653-x)
Supplement: Supplementary file 2 — Supplementary Material 2 [file 12902_2024_1653_MOESM2_ESM.docx]

**Supplementary Table S1.** Baseline characteristics of the males after consideration of SHBG SNPs

| Variables | Total (n=363) | Without malnutrition exposure risk  (n=256) | With malnutrition exposure risk  (n=107) | P |
| --- | --- | --- | --- | --- |
| Age, years | 52.50±12.38 | 52.45±11.97 | 52.62±13.37 | 0.909 |
| Diabetes duration, months | 72.00 (6.00-132.00) | 60.00 (6.00-120.00) | 96.00 (5.00-144.00) | 0.093 |
| SHBG, nmol/L | 34.15±19.06 | 28.94±13.43 | 46.62±24.16 | <0.001 |
| HbA1c, % | 9.92±2.68 | 9.49±2.34 | 10.97±3.16 | <0.001 |
| C-peptide, nmol/L | 0.54 (0.31-0.83) | 0.63 (0.40-0.94) | 0.32 (0.20-0.57) | <0.001 |
| Uric acid, μmol/L | 395.24±105.40 | 409.28±101.00 | 362.48±108.63 | <0.001 |
| Hemoglobin, g/L | 140.01±18.05 | 143.46±15.95 | 131.69±20.08 | <0.001 |
| IGF-1, ng/mL | 160.57±73.74 | 168.17±70.44 | 141.89±78.54 | 0.002 |
| AST, U/L | 19.00 (16.00-24.00) | 20.00 (16.00-24.00) | 19.00 (15.00-25.00) | 0.408 |
| ALT, U/L | 20.00 (15.00-28.00) | 21.00 (15.00-30.00) | 17.00 (13.00-25.00) | 0.013 |
| Creatinine, μmol/L | 71.95 (61.66-86.77) | 72.40 (63.30-85.80) | 69.50 (56.70-97.77) | 0.355 |
| eGFR, (mL/min/1.73 m^2^) | 106.06 (82.66-131.15) | 104.81 (84.10-126.50) | 110.83 (75.36-140.40) | 0.391 |
| Triglyceride, mmol/L | 1.42 (0.98-2.01) | 1.54 (1.10-2.13) | 1.10 (0.83-1.55) | <0.001 |
| Cholesterol, mmol/L | 4.88 (4.07-5.69) | 4.87 (4.19-5.62) | 4.96 (3.90-5.93) | 0.943 |
| LDL-C, mmol/L | 3.31±0.91 | 3.30±0.79 | 3.34±1.16 | 0.727 |
| TT, nmol/L | 10.09±6.87 | 10.16±6.99 | 9.89±6.60 | 0.757 |
| TSI | 2.13±1.94 | 2.21±1.97 | 1.94±1.86 | 0.256 |
| Weight, kg | 69.79±12.51 | 73.40±11.73 | 61.16±9.87 | <0.001 |
| Height, cm | 168.65±5.91 | 168.83±5.94 | 168.21±5.86 | 0.366 |
| BMI, kg/m^2^ | 24.48±3.82 | 25.70±3.47 | 21.57±2.96 | <0.001 |
| Albumin, g/L | 38.03±4.16 | 39.66±2.83 | 33.90±4.14 | <0.001 |
| Prealbumin, mg/L | 236.99±60.89 | 253.39±54.51 | 199.08±58.13 | <0.001 |
| Transferrin, mg/dl | 1.98±0.36 | 2.09±0.30 | 1.72±0.35 | <0.001 |
| NAFLD, n (%) | 145 (39.94) | 124 (48.44) | 21 (19.63) | <0.001 |
| CVD, n (%) | 119 (32.78) | 97 (37.89) | 22 (20.56) | 0.001 |
| Hypertension, n (%) | 154 (42.42) | 118 (46.09) | 36 (33.64) | 0.029 |
| Retinopathy, n (%) | 91 (25.07) | 56 (21.88) | 35 (32.71) | 0.030 |
| ABI, n (%) | 77 (21.21) | 57 (22.27) | 20 (18.69) | 0.448 |

Continuous variables are reported as mean±SD for normally distributed variables or as the median (IQR) for skewed variables, while categorical variables are represented as numbers (proportions).

BMI: body mass index; HbA1c: hemoglobin A1c; SHBG: sex hormone-binding globulin; LDL-C: low-density lipoprotein cholesterol; ALT alanine aminotransferase; AST: aspartate aminotransferase; HDL-C: high-density lipoprotein cholesterol; LDL-C: low-density lipoprotein cholesterol; eGFR: estimated glomerular filtration rate; IGF-1: insulin-like growth factor-1; TT: total testosterone; TSI: Testosterone secretion index; NAFLD: non-alcoholic fatty liver disease; CVD: cardiovascular disease; ABI: ankle brachial index; NA: not available

**Supplementary Table S2.** Baseline characteristics of postmenopausal females after consideration of SHBG SNPs

| Variables | Total (n=188) | Without malnutrition exposure risk  (n=128) | With malnutrition exposure risk  (n=60) | P |
| --- | --- | --- | --- | --- |
| Age, years | 61.44±6.62 | 61.57±6.38 | 61.15±7.16 | 0.686 |
| Diabetes duration, months | 120.00 (72.00-204.00) | 120.00 (72.00-180.00) | 120.00 (66.00-240.00) | 0.800 |
| SHBG, nmol/L | 38.55±20.00 | 34.30±17.61 | 47.61±21.87 | <0.001 |
| HbA1c, % | 9.64±2.16 | 9.23±1.92 | 10.55±2.41 | <0.001 |
| C-peptide, nmol/L | 0.68 (0.43-0.94) | 0.73 (0.50-0.96) | 0.55 (0.37-0.88) | 0.005 |
| Uric acid, μmol/L | 363.67±115.10 | 377.45±112.75 | 332.86±115.34 | 0.016 |
| Hemoglobin, g/L | 123.34±16.02 | 127.20±13.50 | 115.12±17.90 | <0.001 |
| IGF-1, ng/mL | 145.53±70.40 | 149.59±75.55 | 137.22±58.13 | 0.266 |
| LDL-C, mmol/L | 3.41±1.07 | 3.37±0.98 | 3.50±1.24 | 0.460 |
| AST, U/L | 19.00 (15.00-24.00) | 20.00 (17.00-26.25) | 16.50 (13.00-21.00) | <0.001 |
| ALT, U/L | 19.00 (13.00-28.00) | 20.00 (15.00-30.50) | 16.00 (10.75-23.25) | <0.001 |
| Creatinine, μmol/L | 56.80 (48.03-72.58) | 56.32 (47.77-68.48) | 58.08 (49.03-83.78) | 0.303 |
| eGFR, (mL/min/1.73 m^2^) | 99.36 (74.72-122.54) | 101.15 (78.99-124.39) | 96.00 (63.61-119.08) | 0.305 |
| Triglyceride, mmol/L | 1.73 (1.19-2.61) | 1.92 (1.23-2.81) | 1.60 (1.14-2.02) | 0.020 |
| Cholesterol, mmol/L | 5.30 (4.25-6.21) | 5.29 (4.30-6.25) | 5.49 (4.15-6.19) | 0.856 |
| Weight, kg | 60.08±9.55 | 63.15±8.31 | 53.53±8.74 | <0.001 |
| Height, cm | 156.77±5.37 | 156.93±5.54 | 156.41±5.01 | 0.533 |
| BMI, kg | 24.41±3.56 | 25.60±2.98 | 21.76±3.35 | <0.001 |
| Albumin, g/L | 36.75±4.62 | 38.76±2.80 | 32.44±4.80 | <0.001 |
| Prealbumin, mg/L | 226.19±65.53 | 246.47±49.57 | 186.77±74.70 | <0.001 |
| Transferrin, mg/dl | 2.01±0.35 | 2.11±0.30 | 1.81±0.36 | <0.001 |
| NAFLD, n (%) | 76 (40.43) | 68 (53.12) | 8 (13.33) | <0.001 |
| CVD, n (%) | 59 (31.38) | 45 (35.16) | 14 (23.33) | 0.103 |
| Hypertension, n (%) | 109 (57.98) | 76 (59.38) | 33 (55.00) | 0.571 |
| Retinopathy, n (%) | 63 (33.51) | 34 (26.56) | 29 (48.33) | 0.003 |
| ABI, n (%) | 45 (21.81) | 28 (21.88) | 13 (21.67) | 0.974 |

Continuous variables are reported as mean±SD for normally distributed variables or as the median (IQR) for skewed variables, while categorical variables are represented as numbers (proportions).

BMI: body mass index; HbA1c: hemoglobin A1c; SHBG: sex hormone-binding globulin; ALT: alanine aminotransferase; AST: aspartate aminotransferase; HDL-C: high-density lipoprotein cholesterol; eGFR: estimated glomerular filtration rate; IGF-1: insulin-like growth factor-1; CVD: cardiovascular disease; ABI: ankle brachial index; NA: not available


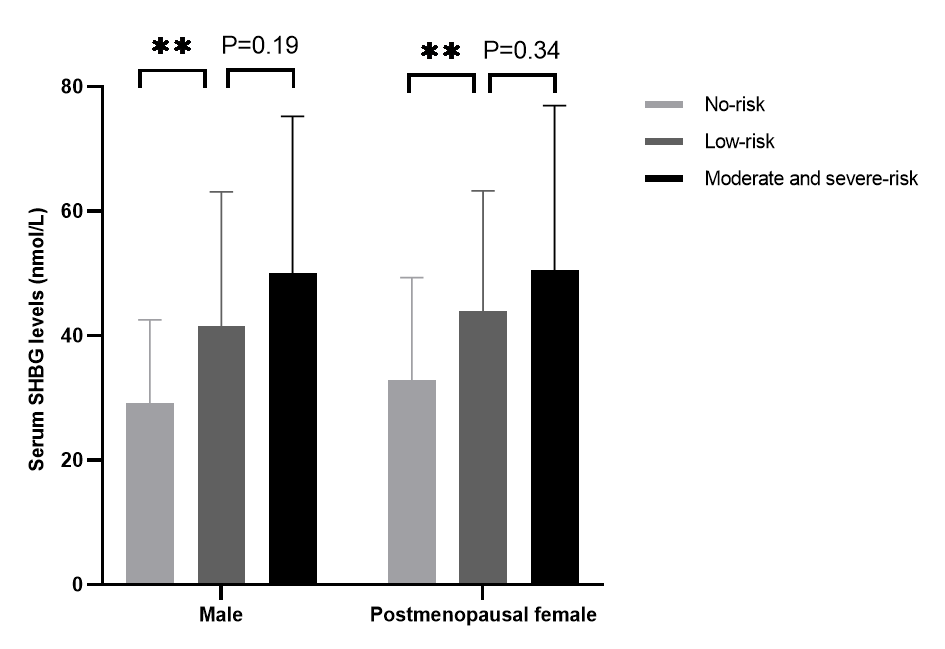


**Supplementary Figure S1.** Subgroup analysis of serum SHBG levels among men and postmenopausal women in no-risk group, low-risk group, and moderate and severe-risk group. * P<0.05, ** P<0.01.
